# Regional wall motion abnormalities at rest and stress in patients with end-stage renal disease diagnosed by cardiac magnetic resonance imaging

**DOI:** 10.1186/1532-429X-11-S1-P267

**Published:** 2009-01-28

**Authors:** Constanze Merten, Christoph Merbach, Vedat Schwenger, Martin Zeier, Hugo A Katus, Evangelos Giannitsis, Henning Steen

**Affiliations:** University Hospital Heidelberg, Heidelberg, Germany

**Keywords:** Coronary Artery Disease, Arterial Hypertension, Dobutamine, Cardiac Magnetic Resonance Imaging, Regional Wall Motion Abnormality

## Introduction

Patients with end-stage renal disease (ESRD) are known to be subject to a high cardiovascular morbidity and mortality mainly due to coronary artery disease (CAD). We sought to assess the prevalence of regional wall motion abnormalities (rWMA) by cardiac magnetic resonance imaging (cMRI) in asymptomatic patients with ESRD.

## Methods

We examined 77 patients with ESRD awaiting kidney transplantation by cMRI using a clinical 1.5 Tesla scanner (Philips Achieva). Steady-state free precession cine images were acquired at rest and during a dobutamine stress test with four dobutamine dose steps from 10 to 40 μg/kg body weight over 3 minutes each. Wall motion was assessed qualitatively by two experienced observers by consensus-reading according to the 17-segment-model. Each segment was classified as normokinetic, hyokinetic, dyskinetic or akinetic.

## Results

Of the examined patients 28 were women (36%), the mean age was 58 years. Diabetes was known in 32 patients (42%), a previously diagnosed CAD in 32 patients (42%). Arterial hypertension was present in 69 patients (90%).

At rest, left ventricular rWMA were present in 38 patients (49%) and 123 segments, respectively. 89 segments were classified as hypokinetic, 24 as akinetic and 10 segments as dyskinetic. In 71 patients a dobutamine stress test with sufficient image quality could be performed, of those 30 patients (42%) displayed rWMA. 63 segments with rWMA were identified at stress thereof 50 with hypokinesia, 12 with akinesia and 1 dyskinetic segment. Among patients with rWMA at rest, 12 showed a normalisation of contractility at stress, in 18 patients WMA remained unchanged during dobutamine stimulation, further 2 patients had worsening WMA at stress. In the remaining 6 patients a stress test could not be performed due to claustrophobic reactions or resulted in insufficient image quality.

In 6 patients (8.5%) a positive stress test was observed. 4 of those patients had normal contractility at rest, in 2 patients pre-existing rWMA worsened under dobutamine stimulation. 3 patients with a positive stress test were diabetic, 2 patients had previously known CAD.

At rest and stress, the prevalence of rWMA was not different in patients with and without diabetes mellitus (47 vs. 51%, p = NS at rest and 45 vs. 40% at stress, p = NS). The presence of arterial hypertension and the myocardial mass equally had no influence of the prevalence of WMA. However, we observed significantly more rWMA in patients with known CAD than in patients with a history of CAD, as well at rest (66 vs. 38%, p = 0.016) as at stress (59 vs. 31%, p = 0.02).

## Conclusion

rWMA are e frequent finding in patients with ESRD. Patients with a known CAD have a significantly higher prevalence of rWMA. However, rWMA were also observed in about a third of patients without a history of CAD. Risk factors such as diabetes mellitus or arterial hypertension did not influence the prevalence of rWMA.

cMRI is a useful and non-invasive method to identify patients with cardiac morbidity in the high risk population of patients with ESRD.

